# Supplementary material for: Lack of Salivary Long Non-Coding RNA XIST Expression Is Associated with Increased Risk of Oral Squamous Cell Carcinoma: A Cross-Sectional Study
Source: J Clin Med. 2021 Oct 8;10(19):4622. doi: 10.3390/jcm10194622 (PMC8509565; doi:10.3390/jcm10194622)
Supplement: Supplementary file 1 [file jcm-10-04622-s001.zip › jcm-1415788-supplementary.pdf]

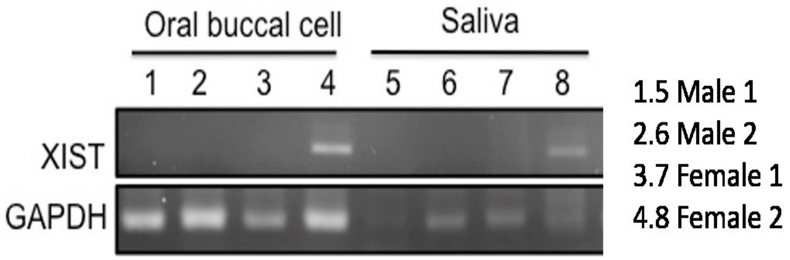

Supplementary Figure S1. The XIST and GAPDH expression of two normal males and two normal females

(A)

OSCC male n=33		Normal male n=16	
XIST	GAPDH	XIST	GAPDH
UD	27.95	UD	30.86
UD	31.04	UD	31.91
UD	26.53	UD	30.34
UD	29.13	UD	31.34
UD	29.58	UD	31.63
UD	29.49	UD	32.49
UD	30.52	UD	31.99
UD	31.28	UD	29.95
UD	28.62	UD	32.82
UD	30.88	UD	28.32
UD	27	UD	30.24
UD	35.82	UD	27.28
UD	31.38	UD	28.96
UD	29.36	UD	34.34
UD	27.69	UD	36.85
UD	28.33	UD	30.75
UD	28.46		
UD	32.2		
UD	33.2		
UD	29.94		
UD	33.61		
UD	28.25		
UD	32.54		
UD	31.96		
UD	29.08		
UD	30.13		
UD	30.42		
UD	25.87		
UD	30.92		
UD	31.23		
UD	29.87		
UD	32.66		
UD	30.98		

(B)

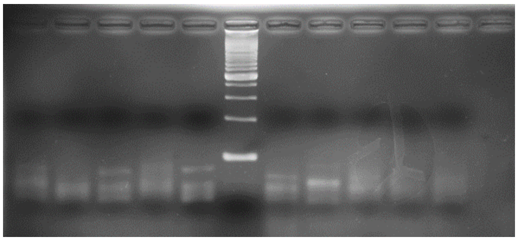

Supplementary Figure S2. The qPCR results of salivary XIST and GAPDH.
(A) The qPCR Ct value of male XIST and GAPDH. UD: undetermined.
(B) Sampling part of the male qPCR sample to Check the XIST expression in agarose gel
